# Microneedling combined with compound betamethasone in treatment of severe alopecia areata: A case report

**DOI:** 10.3389/fimmu.2022.939077

**Published:** 2022-08-03

**Authors:** Danning Pei, Lirong Chen, Yue Yao, Linxi Zeng, Guoqiang Zhang

**Affiliations:** ^1^ Department of Dermatology, The First Hospital of Hebei Medical University, Shijiazhuang, China; ^2^ Candidate Branch of National Clinical Research Center for Skin Diseases, Shijiazhuang, China

**Keywords:** alopecia areata, collapse of hair follicle immune privilege, microneedling, compound betamethasone, dermoscopy

## Abstract

Alopecia areata (AA) is a common inflammatory, non-cicatricial hair loss. At present, it is considered that its pathogenesis is an autoimmune disease specific to hair follicle organs mediated by T cells under the combined action of genetic and environmental factors. Treatment is challenging for children with severe AA who are resistant or intolerant to conventional treatment.Here, we treated a 3-year-old child with severe AA with microneedling combined with compound betamethasone. After 6 months of treatment, the patient’s condition was significantly improved, and most of the primary hair loss areas had hair regeneration.

## Introduction

Microneedling is a relatively new minimally invasive treatment. Microdamage caused by microneedling has been shown to up-regulate the Wnt/β-catenin pathway and induce growth factors and capillaries. This process stimulates the regeneration of hair follicle stem cells and increases the blood supply to the hair follicle, thereby promoting hair growth. Meanwhile, the microchannels formed by microneedling can improve the bioavailability of drugs. At present, microneedling have been found to be successful in promoting hair growth in combination with other hair growth promoting agents such as minoxidil, platelet plasma and topical corticosteroids. So far, the treatment of alopecia with microneedling is mostly studied in androgenetic alopecia, but less in alopecia areata (1).

## Case report

**Figure 1:**
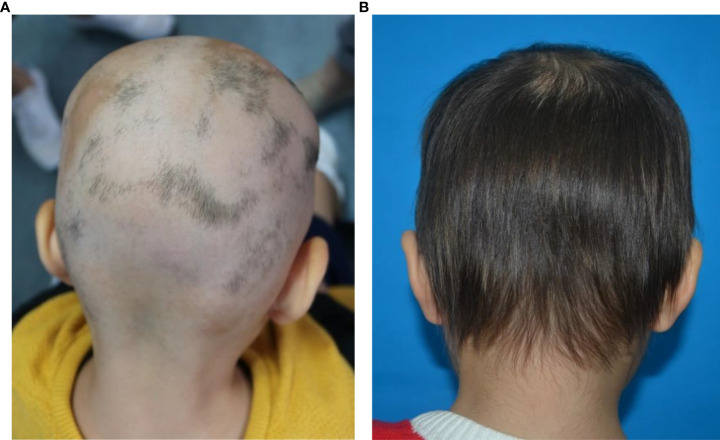
**(A, B)** Clinical pictures show that multiple alopecic patchs on the patient’s occipital scalp before treatment. After 6 months of microneedling treatment, the hair of the patient’s occipital scalp was almost completely regrowth.

The patient is a 3-year-old female. The main reason was “progressive alopecia of the head for 2 months”. Two months ago, after the child was scolded and physically punished by her mother for wetting the bed at night, a coin-sized hair loss appeared on the top of her head in the next morning. The child was treated with oral Chinese medicine at the local clinic for 1 month (the specific treatment is unknown), but her condition was not controlled and her hair was still falling off. Then she was admitted to the dermatology department of our hospital and was treated with topical mometasone furoate for more than 2 months. However, the disease still progressed. Multiple hair loss areas of different sizes appeared in the bilateral temporal, top and occipital parts of the child. Finally, the child only had a small amount of broken hairs remained in the occipital part, and no hair was found in the rest parts (**Figure 1A**). The child was previously healthy and denied having autoimmune diseases and family history.

**Figure 2:**
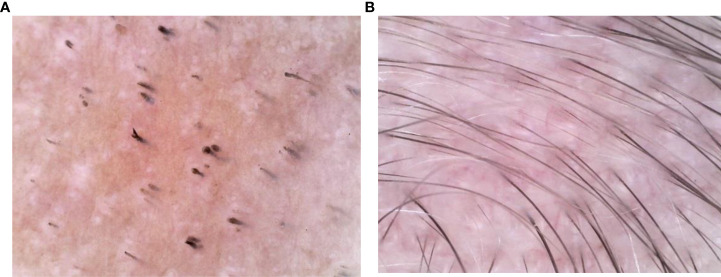
**(A, B)** Dermoscopy show that before treatment, there were yellow spots, black spots, broken hairs and exclamation mark hairs in the patient’s skin lesions. After 6 months of microneedling treatment, the dermoscopic signs of alopecia areata disappeared and 1-2 hairs were visible in each hair follicle.

Dermatological examination: there was almost no hair on the head and temporal part of the child. Several hair loss areas with different sizes and clear boundaries can be seen in the occipital part. Some hair loss areas are fused into pieces, and a small amount of residual broken hairs can be seen at the edge. The skin in the hair loss area is smooth without scales. The child declared no discomfort such as itching or pain in the whole scalp. Except for hair loss, no other body hair loss was found. Fingers and toenails were not affected. Dermoscopy showed that there were yellow dots, black dots, broken hairs and exclamation mark hairs in the hair loss area of the child (**Figure 2A**). Combined with the clinical manifestations and dermoscopic results, the patient was diagnosed as severe alopecia areata.

The patient was treated with microneedling combined with compound betamethasone. Specific process of microneedling treatment: before treatment, compound lidocaine cream was applied to the scalp for 1 hour in order to eliminate the pain during treatment, and then 75% alcohol was used to disinfect the scalp for 3 times. After that, the disposable sterile microneedling infiltration machine was then used for point-to-point operation perpendicular to the scalp surface. At the same time, compound betamethasone injection was applied to the corresponding scalp area for penetration, and massaged with fingers to promote drug absorption. Repeated the above operation for 3 times until the scalp was slightly red. The frequency of microneedling treatment for the child is once a week. The treatment duratione was 6 months. There was no other drug treatment except microneedling combined with duration penetration. After 6 months of treatment, she had almost complete hair regrowth. During the 6 months of microneedling treatment, there were no adverse reactions and no recurrence, and the patient is still being followed up.

## Discussion

Alopecia areata (AA) is an inflammatory and non-cicatricial hair loss disorder. The disease can occur at any age, and its global prevalence is about 2% (2). However, the percentage of children with AA in all age groups is about 25% (3). The etiology and pathogenesis of AA are not completely clear. At present, it is considered that its pathogenesis is an autoimmune disease specific to hair follicle organs mediated by T cells under the combined action of genetic and environmental factors. The collapse of hair follicle immune privilege plays an important role in the pathogenesis of AA. Some nonspecific irritant, such as infection, local trauma, mental or physical stress, can cause the release of large amounts of inflammatory factors such as IFN-γ and TNF-α. These inflammatory factors can induce abnormal expression of MHC-I molecules in hair follicles during growth period and exposure of hair follicle autoantigen, resulting in loss of hair follicle immune privilege. CD8+T cells recognize and destroy hair follicle epithelial cells, and CD4+T cells provide an auxiliary role for them. Eventually, these leads to hair loss (4). The typical manifestation is a sudden round or oval hair loss area with different sizes and numbers and clear boundaries, and the skin of the affected area is normal. Most patients have no obvious symptoms, and a few patients may have itching, tenderness, and scalp tightness. Some patients may have nail changes, such as nail point depression, point white nail and nail longitudinal ridge. Some patients may be complicated with autoimmune or inflammatory diseases, such as Hashimoto’s thyroiditis, lupus erythematosus, atopic dermatitis and vitiligo. Typical AA patients can be diagnosed based on clinical manifestations and dermoscopy. Some patients with atypical AA needed to be differentiated from trichotillomania, pseudoalopecia areata, tinea capitis, systemic lupus erythematosus, syphilitic alopecia, androgenic alopecia and congenital alopecia. Clinical types of AA include patchy, diffuse, ophiasis, total alopecia and universal alopecia. The clinical stages are progressive stage and stable stage. Disease activity is assessed by the amount of hair loss, hair pull tests, dermoscopic features such as yellow dots, black spots, broken hairs, exclamation marks. Internationally, the Severity of Alopecia Tool (SALT) is used to evaluate the severity of AA, which also needs to consider the damage of the nail and hair outside the scalp. The disease has a tendency of self-healing. Researches show that 34% to 50% of mild patients can heal themselves within one year, but 14% to 25% of patients continue or progress to total or universal alopecia. The natural recovery rate of total alopecia and universal alopecia is less than 10% (5, 6).

**Figure 3:**
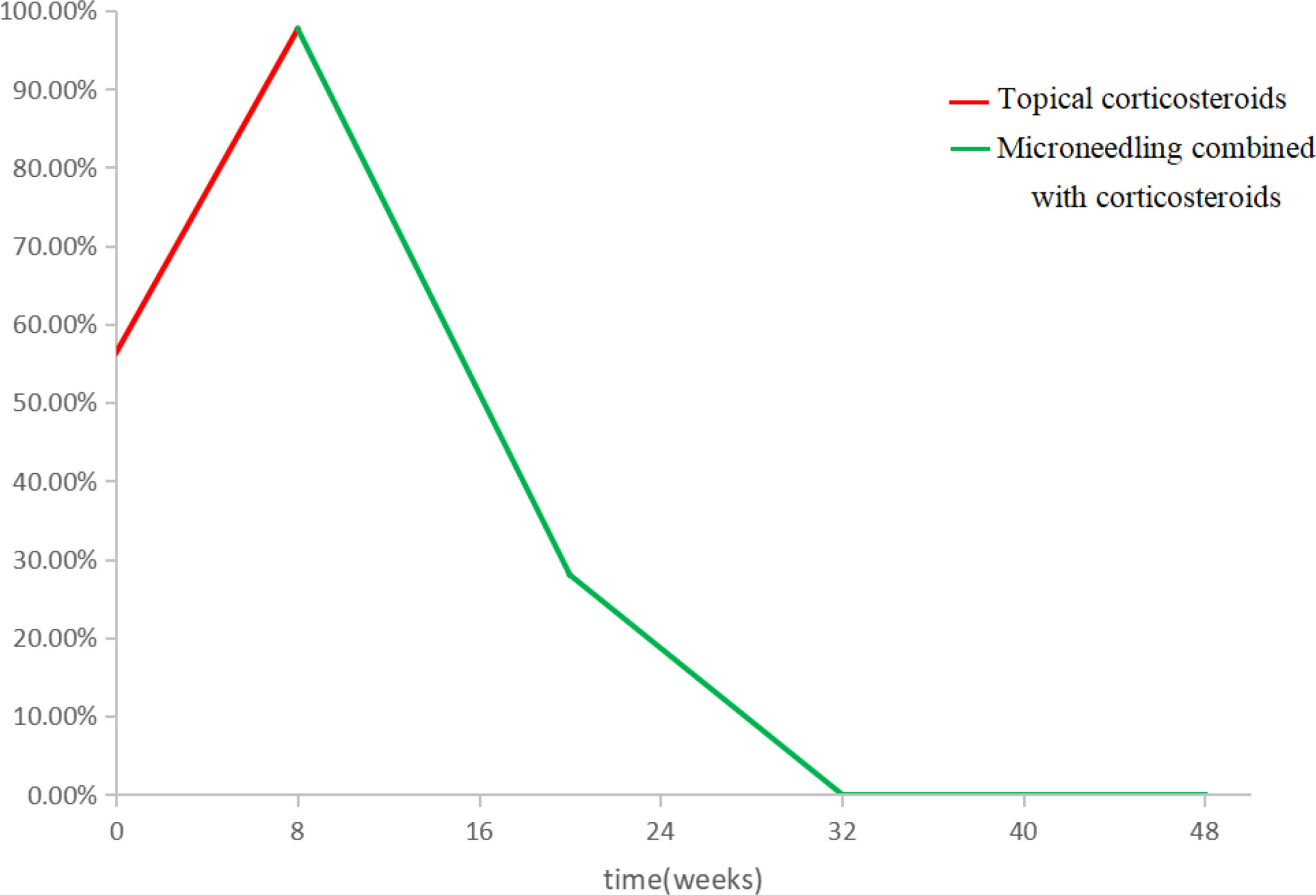
SALT changes before and after microneedle treatment.

The treatment of AA includes topical therapy (topical corticosteroids, calcineurin inhibitors, minoxidil, prostaglandin analogues, contact immunotherapy), intralesional corticosteroids (ILC), systemic therapy (systemic corticosteroids, cyclosporin, methotrexate, Janus kinase inhibitors, azathioprine, mycophenolate mofetil, dapsone, simvastatin/ezetimibe, sulfasalazine, ustekinumab, IL-17A inhibitors, apremilast), phototherapy and Chinese medicine therapy. The Alopecia Areata Consensus of Experts (ACE) study indicates that the choice of treatment is influenced by the patient’s age, disease severity, and course of disease. For children aged 6 years and younger, safety is the primary concern when compared with disease severity and treatment effectiveness. ACE indicats that regardless of SALT, topical corticosteroids are the most appropriate first-line treatment for children under 6 years of age with acute AA. However, when topical corticosteroids are ineffective, there is no consensus on alternative treatment options (7). Since Henry et al. (8) first discovered that microneedling technology can improve the percutaneous absorption rate of drugs by 1000 times, microneedling technology has also become a research hotspot in the field of treating hair loss. In a 12-week randomized, evaluator blind, controlled trial, 100 patients with mild to moderate AGA were randomly assigned to two groups. The experimental group was given microneeding treatment weekly and topical 5% minoxidil twice a day, while the control group was given topical 5% minoxidil only twice a day. The results showed that the hair count per square centimeter in the experimental group was significantly higher than that in the control group at week 12 (91.4 vs 22.2 respectively, P=0.039). At 8 months after the end of the study, all patients in the trial group still had excellent and durable hair growth (9). Another pilot study included 11 female type alopecia with the severity of Ludwig grade I. The scalps of all subjects were randomly assigned: one half of the scalp was treated with growth factor solution five times weekly followed by microneedling (experimental group). The other half of the scalp was treated with normal saline followed by microneedling (control group). At week 5, the hair count in the experimental group increased by an average of more than 10% compared to the control group, and there was a significant difference in the change in hair count between the two groups (52.91 ± 10.85 vs. 45.91 ± 9.98 respectively, P = 0.0001). No adverse events were reported in this study (10). Asad et al. (11) reported a 58-year-old white male with AA, ophiasis pattern, who was treated with 4 treatments of microneedling with triamcinolone over 6 months and showed significant improvement with almost complete hair regrowth in the original hair loss area. Beergouder et al. (12) reported that microneedle combined with triamcinolone acetonide successfully treated an 11 year old female patient with refractory total alopecia. Chandrashekar et al. (13) applied microneedling combined with triamcinolone acetonide to treat 2 patients with refractory AA. After 9 weeks of treatment, obvious curative effect was achieved, and there was no recurrence after 3 months of follow-up. (See **Table 1** for details). The patient in this case was classified as severe AA according to the SALT recommended by the Alopecia Areata Investigational Assessment Guidelines (5). Initially, we followed the guidelines to give topical corticosteroids to patients, but it was ineffective. Based on previous studies, we found that microneeding technology combined with minoxidil, topical corticosteroids and other drugs can be tried as a new method to treat alopecia disease, and its effect and safety are considerable. After a full communication with the parents about microneedle technology, they were very willing to try this new treatment method. On the premise that the parents of the child signed the informed consent and the hospital ethics were passed, we decided to try to use microneedling combined with compound betamethasone penetration therapy to improve the condition of the child. After 6 months of microneedling treatment, the curative effect of the patient is very inspiring. Scalp hair is almost completely regenerated, and the number, color and thickness of newborn hair are basically the same as normal hair (**Figure 1B**). The results of dermoscopy also changed significantly. The original black spots, yellow spots, broken hairs, exclamation mark hairs and other specific indicators of AA in our patients had disappeared, and there were 1-2 healthy hairs in each hair follicle (**Figure 2B**). SALT also improved significantly. The absolute percent scalp hair loss decreased by 56.4% from baseline, and the percent scalp hair regrowth was 100% (**Figure 3**). Compared with previous case reports, our report has the advantage of having the youngest patient and receiving only microneedle combination therapy.

**Table 1:** Reports of microneedling combined with corticosteroids penetration for the treatment of AA.

Author	Age, gender, and medical history	AA type and scale	Microneedling import drug type	Microneedling treatment frequency	Other treatments	Curative effect	Previous treatment
Asad, et al. (11)	58, male, 3 months	ophiasis, moderate	Triamcinolone, 20mg/ml	once every 30 days, 4 times	topical clobetasol 0.05% solution twice daily	At week 24, the hair completely regenerated	–
Beergouder, et al. (12)	11,female, 2 years	alopecia totalis,severe	Triamcinolone, 10mg/ml	once every 20 days, 3 times	minoxidil 2% at night.	At week 12, the hair completely regenerated	a tapering dose of steroids for few days, and oral mini pulse was given for 1 year, but new lesions continued to appear
Chandrashekar B, et al. (13)	female,(age unknown),1 years	multiple patches, mild	Triamcinolone, 10mg/ml	once every 21 days, 3 times	–	At week 6, hair regeneration was about 95%	intralesional injections of triamcinolone acetonide, topical steroid creams, and minoxidil 5% lotion but with no improvement.
Chandrashekar, et al. (13)	male,(age unknown), 6 months	multiple patches, severe	Triamcinolone, 10mg/ml	once every 21 days, 3 times	–	At week 21, the hair completely regenerated	

Dermoscopy is not only limited to the diagnosis of AA, but also has a broad application prospect in the monitoring and follow-up of therapeutic effect of AA. Ganzetti et al. (14) studied the dermoscopic changes of 17 patients with AA after treatment with diphenylcyclopropenone. The results showed that at week 12, compared with baseline, all patients had significantly reduced dermatoscopic features and significantly increased the number of new capillaries, with statistically significant differences. At week 24, 13 patients with complete hair regeneration had complete loss of dermoscopic features. Compared with 12 weeks, vellus hairs increased significantly and was statistically significant. Although our patient’s final dermoscopy indicated that all features of alopecia areata were gone, the limitations of our study are that the appearance and transformation of vellus hairs into terminal hairs were not observed in detail, and the time of disappearance of each microscopic sign of the patient was not recorded.

Hou et al. (15) found that the most common adverse reactions to microneedle therapy were transient erythema and hyperpigmentation after inflammation, and permanent and severe adverse events were rare. However, the limitations of microneedling therapy are the lack of long-term efficacy and safety studies. In addition, the depth of microneedle therapy remains controversial. Faghihi et al. (16) found that average hair count (P = 0.017) and hair thickness (P= 0.007) in the 0.6 mm depth microneedle combined with minoxidil were significantly increased compared with the minoxidil monotherapy group. A penetration depth of 0.6mm is more beneficial than a depth of 1.2 mm. Ro et al. (17) believed that a microneeding depth of 0.5mm seemed to be more effective than 0.3mm. Therefore, in the future, more researches are needed to standardize the depth of microneedling treatment, drug dose, frequency and duration of microneedling treatment, so as to develop the best microneedling treatment plan.

It is promising to use microneedling technology to improve AA patients who are tolerant to conventional treatment. However, the main limitations of our study are that it is a case report and the follow-up period is short. This study could only observe the efficacy and safety of microneeding therapy in this case in a short period of time. In the future, follow-up of this patient will be required and a large number of multicenter, double-blind, randomized controlled trials will be required to demonstrate the long-term efficacy and safety of microneedle therapy. This is more conducive to promote the application of microneedle technology in alopecia diseases, so that the majority of alopecia areata patients benefit.

## Data availability statement

The original contributions presented in the study are included in the article/supplementary material. Further inquiries can be directed to the corresponding author.

## Ethics statement

The studies involving human participants were reviewed and approved by The First Hospital of Hebei Medical University. Written informed consent to participate in this study was provided by the participants’ legal guardian/next of kin. Written informed consent was obtained from the individual(s), and minor(s)’ legal guardian/next of kin, for the publication of any potentially identifiable images or data included in this article.

## Author contributions

DP has made substantial contributions to the conception and design of the work; DP and LC have made substantial contributions to the acquisition, analysis, and interpretation of data for the work; YY and LZ drafted the work and revised it critically for important intellectual content; GZ provides approval for publication of the content; GZ agrees to be accountable for all aspects of the work in ensuring that questions related to the accuracy and integrity of any part of the work are appropriately investigated and resolved. All authors contributed to the article and approved the submitted version.

## Conflict of interest

The authors declare that the research was conducted in the absence of any commercial or financial relationships that could be construed as a potential conflict of interest.

## Publisher’s note

All claims expressed in this article are solely those of the authors and do not necessarily represent those of their affiliated organizations, or those of the publisher, the editors and the reviewers. Any product that may be evaluated in this article, or claim that may be made by its manufacturer, is not guaranteed or endorsed by the publisher.
